# Estimated cost for cardiovascular disease risk-based management at a primary healthcare center in Nepal

**DOI:** 10.1186/s41256-020-0130-2

**Published:** 2020-01-29

**Authors:** Anu Aryal, David Citrin, Scott Halliday, Anirudh Kumar, Prajwol Nepal, Archana Shrestha, Rachel Nugent, Dan Schwarz

**Affiliations:** 1 Nyaya Health Nepal, Kathmandu, Nepal; 2 Department of Global Health, University of Washington, Seattle, WA USA; 3 Department of Anthropology, University of Washington, Seattle, WA USA; 4 Henry M. Jackson School of International Studies, University of Washington, Seattle, WA USA; 5 Icahn School of Medicine at Mount Sinai, Arnhold Institute for Global Health, New York, NY USA; 6 Department of Medicine, NYU Langone Health, New York, NY USA; 7 Gillings School of Public Health, University of North Carolina, Chapel Hill, NC USA; 8 School of Medical Sciences, Kathmandu University, Dhulikhel, Nepal; 9 Center for Methods in Implementation and Prevention Science, Yale School of Public Health, New Haven, CT USA; 10 Department of Chronic Disease Epidemiology, Yale School of Public Health, New Haven, CT USA; 11 RTI International, Seattle, WA USA; 12 Department of Medicine, Division of Global Health Equity, Brigham & Women’s Hospital, 75 Francis Street, Boston, MA 02115 USA; 13 Department of Medicine, Beth Israel Deaconess Medical Center, Boston, MA USA; 14 Department of Medicine, Harvard Medical School, Boston, MA USA; 15 Ariadne Labs, Brigham and Women’s Hospital and Harvard T.H. Chan School of Public Health, Boston, MA USA

**Keywords:** Health care economics and organizations, Cardiovascular diseases, Risk management, Global health, Primary care, Nepal

## Abstract

**Background:**

Low- and middle-income countries are facing an increasing burden of disability and death due to cardiovascular diseases. Policy makers and healthcare providers alike need resource estimation tools to improve healthcare delivery and to strengthen healthcare systems to address this burden. We estimated the direct medical costs of primary prevention, screening, and management for cardiovascular diseases in a primary healthcare center in Nepal based on the Global Hearts evidence based treatment protocols for risk-based management.

**Methods:**

We adapted the World Health Organization’s non-communicable disease costing tool and built a model to predict the annual cost of primary CVD prevention, screening, and management at a primary healthcare center level. We used a one-year time horizon and estimated the cost from the Nepal government’s perspective. We used Nepal health insurance board’s price for medicines and laboratory tests, and used Nepal government’s salary for human resource cost. With the model, we estimated annual incremental cost per case, cost for the entire population, and cost per capita. We also estimated the amount of medicines for one-year, annual number of laboratory tests, and the monthly incremental work load of physicians and nurses who deliver these services.

**Results:**

For a primary healthcare center with a catchment population of 10,000, the estimated cost to screen and treat 50% of eligible patients is USD21.53 per case and averages USD1.86 per capita across the catchment population. The cost of screening and risk profiling only was estimated to be USD2.49 per case. At same coverage level, we estimated that an average physician’s workload will increase annually by 190 h and by 111 h for nurses, i.e., additional 28.5 workdays for physicians and 16.7 workdays for nurses. The total annual cost could amount up to USD18,621 for such a primary healthcare center.

**Conclusion:**

This is a novel study for a PHC-based, primary CVD risk-based management program in Nepal, which can provide insights for programmatic and policy planners at the Nepalese municipal, provincial and central levels in implementing the WHO Global Hearts Initiative. The costing model can serve as a tool for financial resource planning for primary prevention, screening, and management for cardiovascular diseases in other low- and middle-income country settings globally.

## Background

Cardiovascular disease (CVD) is the leading cause of disability and death globally, accounting for 14.8% of disability adjusted life years [1, 2]. Low- and middle-income countries disproportionately bear this burden [3]. In Nepal, CVD is the number one cause of death, accountable for approximately a quarter of deaths in 2015 [1]. The Government of Nepal has committed to achieve a 25% reduction in mortality from non-communicable diseases (NCDs) including CVD, cancer, diabetes, and chronic respiratory diseases by 2025 [4]. Another goal is to prevent ischemic heart disease and stroke by managing 50% of eligible patients with medicines and counselling [4].

Nepal has been adopting the World Health Organization’s (WHO) Package of Essential Noncommunicable disease interventions (PEN) for primary care in low-resource settings [3], and participates as a pilot site for the WHO Global Hearts Initiative [5] in order to meet the target goals. These programs recommend using a score developed by the International Society of Hypertension, which is calculated based on age, sex, blood pressure, smoking status, and fasting blood glucose and cholesterol [6]. This is an absolute risk score that estimates the likelihood of someone developing a cardiovascular event over a 10-year period [6]. Individuals may fall into any of four risk categories: < 10%, 10–20%, 20–30, and > 30% [6]. Those classified as first category (< 10%) receive only behavioral counselling on healthy lifestyle, and are followed up after one year. Individuals at higher risk are treated with medicines and counselled on healthy lifestyle, with increased intensity and follow up [6] (Additional file 1).

Despite the Government of Nepal acknowledging CVD as a major priority, there are no local data on the cost of providing risk score-based CVD primary prevention and management services at the primary level. Costing analyses can assist policymakers and program planners in estimating care delivery costs and programmatic logistics including: human resources, essential medicine formularies, supplies, and laboratory tests (see Methods>Human resources below for a detailed description of human resources in the context of primary CVD prevention and management in Nepal). This study estimates the annual, direct medical costs of providing risk-based, primary CVD prevention, screening and management according to the WHO Global Hearts Initiative at a hypothetical primary healthcare center (PHC) in Nepal, serving a population of 10,000, at 50% coverage.

## Methods

### Setting

The setting for this study is a hypothetical primary healthcare center (PHC) in Nepal, which exist at scale throughout the country’s 75 districts. The country’s public health system delivers health care services through primary, secondary and tertiary-level healthcare services and facilities. The primary level provides basic minimum health care and serves as a first point of contact; this care is delivered via networks of community health workers and at health-posts and PHCs. The secondary level includes hospitals, which serve as referral units to the primary level, and the tertiary level includes specialty hospitals and other higher referral centers. There are over 200 PHCs in Nepal, each staffed with at least one medical officer (MBBS physician), a staff nurse, and several mid-level providers.

### Costing model

We adapted the CVD-diabetes treatment cost sheet from the WHO NCD Costing Tool [7], an excel file that projects costs for scaling comprehensive NCD programs nationally (see Additional file 1 for costing model). The WHO NCD Costing Tool was developed as part of the PEN interventions tools for low-resource settings [7, 8]. While this tool is useful to forecast financial resources at a national and sub-national level, we adapted the tool to predict the costs of primary CVD prevention, screening, and management at the primary healthcare center level, which required only descriptive information about the catchment area population and the applicable unit costs.

We estimated the direct costs for implementing CVD ‘risk-based management’ as outlined in the Global HEARTS Technical package for CVD management at a PHC in Nepal [9]. We defined direct costs as costs related to service provision including: human resources, medicines, and laboratory tests. We included costs for screening, treatment, and follow up for patients presenting at a hypothetical PHC in Nepal (see Additional file 1 for costing model). We thus calculated the costs strictly from the perspective of the public sector healthcare system (i.e. a PHC managed by the Ministry of Health and Population) and have not included patient-specific costs such as transportation, caregivers, and lost income.

The input parameters and output estimates of the model are listed in Table 1. The model estimated annual cost per case, cost for the entire population, and cost per capita. It also estimated the amount of medicines for one-year, annual number of laboratory tests, and the monthly incremental work load of physicians who deliver these services.

**Table 1:** Costing model input and output parameter estimates

Input (parameters to enter in the model)	Output (parameters estimated from the model)
• Unit price of medicines	• Annual cost per case
• Unit cost of laboratory tests	• Annual cost for the entire population
• Monthly salary of clinicians	• Annual cost per capita
• Service utilization rates	• Amount of different medicines needed in a year
• Coverage rates	• Monthly incremental work load of physicians (time)
• Population demographics	

### Services

The PHC, a municipal-level service unit, was chosen for this study because the HEARTS Technical package is designed for such primary healthcare delivery settings. We estimated the catchment area population of a PHC at 10,000 and used the treatment protocols from the HEARTS Technical package [9] (Additional file 1) to assign services and medicines needed for each risk category. According to the protocol, a physician assesses the patient’s medical history and conducts a clinical examination upon arrival at the PHC. Suspected or known cases of ischemic heart disease are referred to a higher center, while the rest of the patients are sent for laboratory tests (costs associated with these referral cases are not included in this costing study). Each CVD risk score was then calculated based on the medical examination and laboratory test results [6]. Medical treatment was provided to the individuals with a risk score of ≥10%, while only behavioral counselling is provided for those with a risk score of < 10%.

### Target population

We calculated the age and sex distribution of the population using National Population and Census 2011 data [10]. Globally, several guidelines suggest routine cardiovascular risk assessment for people aged 40 years and above, thus we focused on that population in our estimation [11–15]. We estimated the proportion of adults 40 years of age and older, with at least one NCD risk factor, as identified in the Nepal STEPS survey 2013 [16]. We assumed the center’s population coverage to be 50%, which was the coverage goal set by the Government of Nepal’s targets in the Multisectoral Action Plan for the Prevention and Control of Non Communicable Diseases (2014–2020) [4]. We also did a sensitivity analysis with varying coverage of 20, 40, 50, 60, 80, and 100% (Additional file 2). Due to the lack of population-wide CVD absolute risk distribution data from Nepal, we used the population risk profile for WHO South-East Asia Region C for estimates of risk distribution in Nepal [6, 17].

We used the term ‘cases’ to denote all unique individuals arriving at the PHC to receive CVD services, regardless of their risk status. Individuals with risk > 10% enrolled in treatment were considered ‘patients’.

### Costs

We estimated the cost from the perspective of Nepal’s Ministry of Health and Population, as the payer for public PHCs in Nepal. We used a one-year time horizon, presenting costs both in current Nepali rupees (NPR) and current US Dollars (USD).

### Medicine and laboratory tests pricing

To calculate pharmaceutical and laboratory service prices, we used the standard rates made available by the Health Insurance Board [18]. The Board published a standard rate list of medicines and services, on the basis of which they reimburse the healthcare facilities, regardless if they are a PHC or a tertiary hospital, for the services they provide to an insured patient. We assume that administration and supply chain costs for medicines and laboratory have been included when determining these rates by the board, and thus we have not included such additional costs in our study. The HEARTS Technical package suggested the use of two types of statins: either Simvastatin, or Atorvastatin. We used only Atorvastatin in our study because it is one of the drugs in the standard rate list. We consulted with a WHO representative to determine the daily average dose of the drugs for costing purposes (AA personal correspondence, March 22, 2017).

### Human resources

At least one physician (known locally as medical officers; physicians holding only a Bachelor of Medicine, Bachelor of Surgery degree and no postgraduate medical education training) and at least one nurse (nurses holding either a 4 year Bachelor of Science in Nursing degree or a 3 year Proficiency Certificate Level diploma) are assumed to be employed at the PHC as is consistent with the Ministry of Health and Population’s staffing patterns [19]. Actual staffing patterns at PHCs vary and may include auxiliary nurse midwives, health assistants, and other auxiliary health workers who are presumed not to be involved in primary CVD prevention, screening, or management per the Global Hearts protocol (see above section Methods>Services) [19]. We assumed physicians spend 5 minutes for first-visit medical examinations and 5 minutes for interpreting laboratory results and risk profiling. Each physician’s encounter for subsequent patient visits was assumed to be 5 minutes and the nurse’s encounter with the patient for counselling sessions was also assumed to be 5 minutes. This encounter time was consistent with findings from a systematic review which reported that 50% of the global population spent on an average 5 minutes or less time with their primary care physicians during each encounter [20].

We used the Ministry of Health and Population’s healthcare professionals’ salary scale for human resource costs of physician and nurses. A working year of 240 working days, and 40 h per week, as per the Government calendar, was used to calculate per minute price of human resources.

## Results

Laboratory test costs at PHCs are listed in Table 2 in both NPR and USD.

**Table 2:** Laboratory test costs

Laboratory tests	Cost per test^b^	
	NPR	USD
Urine routine test^a^	40	0.39
Random blood glucose	60	0.58
Urine ketone	50	0.48
Serum creatinine	100	0.97
Total cholesterol	150	1.45
Serum potassium	150	1.45

^a^Urine routine test includes urine glucose and protein test

^b^1 USD = 103.36 NPR

The average daily dose and costs for medicines are listed in Table 3. Atorvastatin was found to be the most expensive drug among others, at USD0.10 per day.

**Table 3:** Daily cost of WHO Global Hearts medicines

Drugs	Average daily dose (mg)	Cost per day^a^	
		NPR	USD
Medicines for high blood pressure			
Amlodipine	10	7.00	0.07
Atenolol	50	3.50	0.03
Enalapril	10	8.00	0.08
Hydrochlorthiazide	25	2.95	0.03
Medicines for high blood glucose			
Metformin	1500	6.00	0.06
Glibenclamide	10	3.46	0.03
Insulin Isophane	24(IU)	1.51	0.02
Medicine for high cholesterol			
Atorvastatin	10	10.77	0.10

^a^1 USD = 103.36 NPR

Table 4 shows the incremental increase in time required for healthcare providers in delivering these services. In a population of 10,000, we estimate that the total number of patients needing primary CVD management (24.67%) over age 40 with at least one risk factor (70.12%) at 50% coverage will be 865. Of those 865 patients, 50 (5.7%) will need a referral to a tertiary center further CVD management (see Additional file 1 for population model). At 50% coverage, with a PHC serving a population of 10,000, an average physician’s workload will increase annually by 190 h for physicians and by 111 h for nurses, i.e., additional 28.5 workdays for physicians and 16.7 workdays for nurses.

**Table 4:** Incremental time of healthcare providers in CVD management cases

Clinical encounter	Time per case per year (minutes)		Patients needing services annually	Time to treat the population per year (minutes)	
	Physician^a^	Nurse		Physician	Nurses
First visit-clinical exam	5	0	865	4325	0
First visit-risk profiling	4	0	815	3264	0
Management for risk < 10%	0	5	566	0	2832
Management for risk 10–20% with high blood pressure	10	10	60	600	600
Management for risk 10–20% with high cholesterol	10	10	60	600	600
Management for risk 20–30%	10	10	52	520	520
Management for risk > 30%	20	20	77	1540	1540
Management of diabetic patients	20	20	29	580	580
Annual incremental time (minutes)				11,429	6675
(hours)				190	111
Annual incremental time (workdays)^b^				28.5	16.7

^a^Physician provides medical treatment and nurse conducts counselling sessions

^b^Annual incremental time calculated based on healthcare staff members working 40 h per week and 6 days a week

In Table 5, we show the annual cost of treating cases by different risk types. The cost for a medical examination and risk profiling during the first visit is USD2.49 per case per year. The cost to treat patients with higher risk is greater, as people with higher risk need more frequent follow up and more intense medical treatment. There are also additional medicine costs for treating diabetic patients.

**Table 5:** Annual cost of treatment disaggregated by service type

Services	Physician’s encounter	Laboratory Tests^a^	Counselling^a^	Drugs^a^	Annual cost per case^b^	
					NPR	USD
First visit-clinical exam	26.26	0	0	0	26.26	0.25
First visit-risk profiling	21.01	210.25	0	0	231.26	2.24
plus additional cost for management of patients in each risk categories						
risk < 10%	0	0	19.10	0	19.10	0.18
risk 10–20% with high blood pressure	52.52	0	38.20	3149.04	3239.76	31.34
risk 10–20% with high cholesterol	52.52	400	38.20	3931.05	4321.77	41.81
risk 20–30%	52.52	760	38.20	7463.34	8054.06	77.92
risk > 30%	105.04	1160	76.40	7463.34	8444.78	81.70
plus additional cost for						
Diabetes management	79.28	780	76.40	5413.06	6194.50	59.93

^a^All prices are in NPR unless mentioned otherwise

^b^1 USD = 103.36 NPR

Table 6 provides the annual cost of treatment at the population level for a PHC serving a catchment area of 10,000 people at 50% coverage rate. We estimate 2 million rupees (USD18,621) are needed to provide direct medical services to the entire catchment area based on the HEARTS Technical package. The average total cost per case per year is USD21.53, and the cost per capita per year is estimated to be USD1.86.

**Table 6:** Annual cost of treatment for a population of 10,000 people at 50% coverage

Services and treatment	Unit cost (NPR)	Target Population	Total Cost	
			NPR	USD
First visit-medical examination	26.26	865	22,715	220
First visit-risk profiling	231.26	815	188,708	1826
risk < 10%	19.10	566	10,830	105
risk 10–20% with high blood pressure	3239.76	60	194,386	1881
risk 10–20% with high cholesterol	4321.77	60	259,306	2509
risk 20–30%	8054.06	52	418,811	4052
risk > 30%	8444.78	77	650,248	6291
Management of diabetic patients	6194.50	29	179,641	1738
Total cost			1,924,645	18,621

1 USD = 103.36 NPR

When the total annual cost is disaggregated, medicines cause the largest additional cost (80%), followed by laboratory tests (15%). Doctor’s office visit and counselling from nurses are responsible for a relatively smaller share of cost in the total direct medical cost i.e., 3 and 2% respectively.

## Discussion

For a PHC with a catchment population of 10,000, the estimated cost to screen and treat 50% of eligible patients according to the WHO Global Heart Initiative risk-based primary CVD management was USD21.53 per case, USD1.86 per capita, and USD18,621 in total per annum. The cost of a medical exam and risk screening eligible people for CVD is USD2.49 (NPR 257.52) per person in Nepal. We estimated that a PHC with a catchment population of 10,000 will require USD18,600 annually to provide medicines, laboratory analysis, and human resources for primary CVD prevention, screening, and management services to 50% of the eligible population.

The treatment cost per patient is higher for patients with higher risk scores. A study in Tanzania reported the incremental cost to treat patients with a risk of > 30% to be almost double the cost to treat patients with a risk of 10–20%, and is consistent with our findings as well [21]. Notably, however, the Tanzanian cost estimates were significantly higher than the estimates we have calculated here, which may be due to regional cost differences in service availability, supply chains, or patient-related utilization. Hence, early screening and management can be a key strategy to prevent patients from moving into higher risk categories and incurring larger costs. Early intervention has been shown to lower the costs of services compared to treating patients with higher risk of CVD-related morbidity in developing countries [22].

The estimated annual cost per case is USD21.50, while the per capita cost is USD1.86. Our results are similar to another study analyzing the per capita cost for risk-based management in low-income countries [23]. A previous WHO study estimated the cost of screening to be USD3.00 per person (USD3.40 per person when adjusted to 2018 rates) in low-income countries [8]. Our study demonstrated that a PHC with a catchment population of 10,000 will require USD18,600 annually to provide medicines, laboratory analysis, and human resources for primary CVD prevention, screening, and treatment services to 50% of the eligible population.

The costs estimated here must be considered in the context of local healthcare spending. The Government of Nepal currently spends USD10.12 per capita in healthcare expenditure [24], thus, an increase of USD1.86 per capita would amount to a 18.4% increase in healthcare spending. It is also important to note that only 23% of the total healthcare expenditure is paid by the Government while 60% is out-of-pocket [24], with some out-of-pocket healthcare expenditure estimates for specific locales, medical conditions, and sub-populations being even higher (the remainder is paid via external development assistance sources) [25, 26]. High out-of-pocket healthcare expenditures threaten people with low economic status due to impoverishment. In a recent report, the Nepal Noncommunicable Diseases and Injuries Poverty Commission reported that NCDs and injuries account for 33% of out-of-pocket healthcare expenditure by Nepalese patients [27]. Catastrophic healthcare expenses for secondary CVD treatment and the resulting impoverishing effect have already been noted in many low- and middle-income countries including Nepal [28]. Given this relationship, the Lancet Taskforce on NCDs and Economics recommends removing financial barriers to the poor to improve healthcare systems financing [28]. This makes a case for the Government of Nepal to increase spending on primary CVD prevention, screening, and management strategies to reduce the disease burden, as well as to protect the poor from economic hardship and financial shock [29].

While our study is purely based on theoretical modeling, some insights arise from this work. In particular, total costs will vary by coverage level. The total annual cost would be about USD6,000 at a 20% coverage rate, and about USD24,000 for an 80% coverage rate. One strategy for the Government of Nepal is to reduce overall costs at higher coverage and to raise the current cut-off risk level for treatment to > 20% [6]. Increasing the threshold for treatment would reduce costs overall but could result in lower healthcare benefits [30]. The cost savings might then be short-term, as future costs relating to treating complications of unidentified high-risk patient could even be higher.

Investing in primary CVD prevention, screening, and management utilizing a risk-based approach is a highly cost-effective intervention [23, 31, 32]. Investing in primary prevention can be a cost saving strategy as treating acute and post-acute ischemic heart disease are more expensive [32]. In Nepal, myocardial infarction management costs USD435, which is 234 times the cost of primary CVD prevention, screening, and management as calculated in our study [18]. Similarly, management of diabetes complications costs USD155 per person, compared to only USD 60 for primary diabetes management [18]. The Government of Nepal currently provides free treatment for heart diseases to senior citizens over 75 years [33]. Therefore, investment in early prevention can potentially reduce the Government’s long-term expenditures for treatment of heart disease in the elderly. Cost savings would also result from a potential reduction in mortality, as a high proportion of the NCD burden lies in working age adults [3]. Nugent and Brouwer estimated that the expected return on investment of the CVD risk-based management intervention is USD34 for every USD1 spent on hypertension management for a medium- to high-risk patient at 50% coverage [34]. Reaching a level of 50% coverage of primary CVD prevention, screening, and management in low-income countries is expected to bring 2.3 times in economic return and 3.8 times in economic and social return [35]. This means investing USD1.86 per capita in primary CVD prevention, screening, and management can bring in USD4.3 per capita in economic return, and USD7 in economic and social return. If projected for Nepal’s total population of 29 million, this would require an investment of USD54 million and yield an economic return of USD124 million. Thus, investing in primary CVD prevention, screening, and management could be a win-win situation for Nepal with reductions in premature mortality as well as the potential for long-term economic returns.

Our study estimated that the direct cost of management of type 2 diabetic patients is USD60 per annum. A cross-sectional study in a public hospital in Nepal estimated the direct cost to range from USD54 to USD113 annually [36]. Such a large range in cost is likely because of large variation in the cost of medicines in their study, which ranged from USD18 to USD66, and accounted for more than 80% of direct medical costs. The study also included transportation costs, food during hospital visits, and in some cases the cost of lifestyle modification services. We did not include those costs in our study, as our study estimated the cost from the Government of Nepal’s perspective.

The cost of medicines was the largest driver of costs for each level of risk management, as is consistent with other studies [23, 31, 36]. The National Health Accounts report of Nepal indicates that almost 40% of current health expenditure costs are pharmaceutical expenditures [37]. This means that variability in drug prices can have a higher impact on variation of overall cost. One measure to control the pharmaceutical price could be fixing the maximum retail price of medicines, as is being currently done by the National Health Insurance Board of Nepal [18].

It is also important to note that the program incurs not only additional costs, but puts a demand on the existing healthcare system as a whole. Currently PHCs in Nepal have one physician and one staff nurse employed full time, along with other non-physician healthcare workers. If this program is to be implemented, there is an additional workload for providers. There is also a need to strengthen the laboratory, as this intervention can potentially require additional laboratory services for 865 people annually. We estimate that the program will also need around 200,000 units (tablet/capsule/vial) of medicines annually, which means logistic management capacity has to be increased, as well. Linking patients to chronic care also means ensuring a continuous supply of medicines, which is difficult given that many Nepalese healthcare facilities are unable to maintain stocks of medicines throughout the year [38].

There are six major limitations of our study. The first major limitation is that we have only included incremental costs in terms of direct medical cost including: human resources, laboratory tests, and treatment. We have not included capital costs, assuming sufficient PHC infrastructure. Many actual PHCs may need infrastructure upgrades, strengthening of administrative services, and human resource training prior to initiating this program, which is not currently captured in our study. Second, we heavily relied on assumptions on coverage, adherence, providers’ time, and service utilization rates to develop the cost estimates as the program has not yet been implemented. In future research studies, using actual patient data including costs, utilization, time allocated per encounter, and other real-world details will provide more accurate insights into these questions. Third, we modelled our study with an ideal scenario where all patients are followed through the patient care pathway model. In actual practice, healthcare systems may lose some patients in each of the treatment steps from screening to treatment to follow-up. Fourth, we assumed the availability of medicines and services match with the needs of the population, but many of the healthcare facilities in Nepal struggle with scarcity of trained human resources and medical supplies for NCDs [38]. We acknowledge that some PHCs, especially in remote places of Nepal, may not have a physician, but only have non-physician healthcare workers. If needed, the user of the costing model can customize the human resource section to adjust to different local contexts. Fifth, we are not able to capture costs from the patient’s perspective, which includes the costs of transportation, caregivers, lost income, etc. These additional costs may be substantial, can impede patients from seeking care, and are important to understanding the total societal cost of CVD management services. And sixth, our model only accounts for costs associated with western biomedical care and not alternative therapeutics obtained through non-Western providers [39].

A prospective costing study during the program’s actual implementation is needed to overcome these limitations. A costing tool developed to accompany the Global Hearts technical package is currently being pilot tested in several other countries with support from the US Centers for Disease Control. Future studies may also include other systems-level costs like human resources training, logistic management, and administrative costs in addition to direct medical costs. The next step is to implement and refine this model in a care delivery setting.

## Conclusions

PHCs in Nepal can provide primary CVD risk-based management, screening, and treatment at an average cost of USD21 per case or USD2 per capita. This is the first costing study for a PHC-based, primary CVD risk-based management program in Nepal, which can assist program planners at the Nepalese municipal, provincial and central levels in implementing the WHO Global Hearts Initiative. This method of cost estimation and this costing exercise can also be utilized in other low-resource settings around the world to inform decisions around the affordability of CVD prevention services.

## Supplementary information

**Additional file 1.** CVD costing tool.

**Additional file 2.** Sensitivity analysis.

## Data Availability

The dataset supporting the conclusions of this article is included in Additional file 1.

## Abbreviations

CVD: Cardiovascular disease
NCD: Non-communicable disease
NPR: Nepalese rupee
PEN: Package of Essential Non-communicable Disease Interventions
PHC: Primary healthcare center
USD: United States dollar
WHO: World Health Organization
