# Supplementary material for: Estimated cost for cardiovascular disease risk-based management at a primary healthcare center in Nepal
Source: Glob Health Res Policy. 2020 Jan 29;5:2. doi: 10.1186/s41256-020-0130-2 (PMC6988194; doi:10.1186/s41256-020-0130-2)
Supplement: Supplementary file 2 — Additional file 2. Sensitivity analysis. [file 41256_2020_130_MOESM2_ESM.docx]

**Sensitivity Analysis**

We conducted a sensitivity analysis to see how changes to input parameters affect the output parameters. Here, we changed the coverage rate to see its effects on the workload of healthcare workers. The incremental workload time for healthcare workers varies because this program depends on coverage rate. If the coverage of the program is high, more cases will come into the facility to receive the services, thus physician and nurse work load will correspondingly increase compared to that at lower coverage rate.

Table 1: Annual incremental work load increase for healthcare workers

| Coverage | Physicians^*^ | | Nurses^*^ | |
| --- | --- | --- | --- | --- |
|  | *Hours* | *Work days* | *Hours* | *Work Days* |
| 20% | 76 | 11.4 | 45 | 6.8 |
| 40% | 153 | 23.0 | 89 | 13.4 |
| 50% | 190 | 28.5 | 111 | 16.7 |
| 60% | 228 | 34.2 | 133 | 20.0 |
| 80% | 305 | 45.8 | 178 | 26.7 |
| 100% | 381 | 57.2 | 222 | 33.3 |

*Annual incremental work load based on physicians and nurses who work 6 days a week, and 40 hours per week

In Figure 1, we estimate the total program cost changes with program coverage, keeping everything else constant.

Figure 1: Variation with annual program cost by coverage
